# First record of *Phormia regina* (Meigen, 1826) (Diptera: Calliphoridae) from mummies at the Sant’Antonio Abate Cathedral of Castelsardo, Sardinia, Italy

**DOI:** 10.7717/peerj.4176

**Published:** 2018-01-04

**Authors:** Giorgia Giordani, Fabiola Tuccia, Ignazio Floris, Stefano Vanin

**Affiliations:** 1 School of Applied Sciences, University of Huddersfield, Huddersfield, United Kingdom; 2 Dipartimento di Agraria – Sez. Patologia vegetale ed Entomologia, University of Sassari, Sassari, Italy

**Keywords:** Archeological context, Insect colonization, Mummified bodies, Ancient burial, Mediterranean

## Abstract

The studies of insects from archaeological contexts can provide an important supplement of information to reconstruct past events, climate and environments. Furthermore, the list of the species present in an area in the past allows the reconstruction of the entomofauna on that area at that time, that can be different from the nowadays condition, providing information about biodiversity changes. In this work, the results of a funerary archaeoentomological study on samples collected from mummified corpses discovered during the restoration of the crypt of the Sant’Antonio Abate Cathedral of Castelsardo (Sardinia, Italy) are reported. The majority of the sampled specimens were Diptera puparia, whereas only few Lepidoptera cocoons and some Coleoptera fragments were isolated. Among Diptera, Calliphoridae puparia were identified as *Phormia regina* (Meigen, 1826) and *Calliphora vicina*, (Robineau-Desvoidy, 1830) both species typical of the first colonization waves of exposed bodies. Three puparia fragments were also identified as belonging to a *Sarcophaga* Meigen, 1826, species (Sarcophagidae). Several Muscidae puparia of the species *Hydrotaea capensis* (Weidmermann, 1818), a late colonizer of bodies, and typical of buried bodies were also collected. The few moth (Lepidoptera) cocoons were identified as belonging to the family Tineidae. This family comprises species feeding on dry tissues and hair typical of the later phases of the human decomposition. Among Coleoptera a single specimen in the family Histeridae, *Saprinus semistriatus* (Scriba, 1790) and a single elytra, potentially of a species in the family Tenebrionidae, were also collected. Overall, the samples collected indicated an initial colonization of the bodies in an exposed context, mainly in a warm season. This research allows the finding of elements indicating the presence, at least in the past, of *P. regina* in Sardinia. This species at the moment seems extinct from Sardinia while it is quite common in the continent.

## Introduction

Funerary archaeoentomology is the application of the principles and techniques used in forensic entomology to human and animal remains, tombs, mummies and other burials of archaeological interest (Huchet, 1996; Huchet, 2014). The two disciplines, forensic entomology and funerary archaeoentomology, are separate and well distinct despite sharing the same bulk of knowledge related to insect colonization of bodies and carrions, and using some common techniques for the collection and analysis of the samples. In fact, it does not make any sense referring to forensic entomology (from the latin *forum*, related to the court) in an archaeological context, being the word forensic referable only to a legal mandate. It is worth mentioning that forensic archaeology refers to the application of the archaeological techniques (body search, excavation, etc.) to a forensic context and not *vice versa*.

In an archaeological context, the knowledge of the ecological and biological specificity of the species associated with the remains can be especially valuable to reconstruct the funerary practices (Dirrigl & Greenberg, 1995; Gilbert & Bass, 1967; Huchet & Greenberg, 2010; Nystrom, Goff & Goff, 2005), to describe the cadaver taphonomy (Gaudio et al., 2015; Huchet & Greenberg, 2010; Vanin et al., 2009; Wood, 1976; Wylie, Walsh & Yule, 1987) and to understand the hygienic and social conditions of the investigated human populations (Capasso & Di Tota, 1998; Ewing, 1924; Huchet, 1996; Raoult et al., 2006; Reinhard & Buikstra, 2003; Rick et al., 2002).

If several extrinsic and intrinsic factors can affect the body colonization by insects (for a review see Campobasso & Introna, 2001) in the archaeological framework, the cultural context and the funerary traditions and practices must also be considered to interpret the entomological findings (Huchet, 2014).

Although the presence of mummified or partially mummified human and animal bodies of archaeological interest is reported from all over the world, the most studied from an entomological point of view are from Europe (Portugal, Italy, France, UK) (Couri et al., 2009; Couri et al., 2008; Huchet, 2010; Huchet, 2013; Panagiotakopulu & Buckland, 2012; Vanin, 2016), North Africa (Egypt) (Huchet, 2016; Huchet et al., 2013a; Panagiotakopulu, 2001), and Central and South America (Peru, Mexico) (Huchet & Greenberg, 2010; Nystrom, Goff & Goff, 2005; Reinhard & Buikstra, 2003).

In Europe, the most frequently reported species from archaeological contexts belong to the order Diptera (families: Calliphoridae, Fanniidae, Muscidae, Phoridae and Sarcophagidae) and Coleoptera (families: Anobiidae [Anobiinae and Ptininae], Cleridae, Cryptophagidae, Rhyzophagidae, Dermestidae, Histeridae and Tenebrionidae) (Huchet, 2014; Morrow et al., 2015).

Adult beetles (Coleoptera) are normally well preserved due to their very resistant exoskeleton, whereas adult flies are generally badly preserved in an archaeological context. However the puparia—empty pupal cases resulting from the transformation of the last cuticles of the larvae preceding the metamorphosis—can survive thousands or even millions of years (Gautier & Schumann, 1973; Germonpré & Leclercq, 1994; Handschin, 1944). Puparia represent the main fraction of the insect remains that can be found associated with a cadaver because of their physical and chemical resistance and because of being the external case of the immobile stage of the insect development. Adult fly remains and larval beetle exuviae can also be collected from the archaeological context but they are usually rare and badly preserved.

In this work we illustrate the results of the archaeoentomological studies on samples collected from mummified bodies discovered during the restoration works of the crypt of the Sant’Antonio Abate Cathedral of Castelsardo (Sardinia, Italy). The Church is located in the village of Castelsardo, in Northern Sardinia, one of the two main Italian islands in the Mediterranean Sea. This archaeological work allows us to document, for the first time, the presence in Sardinia of *Phormia regina* (Meigen, 1826) which is no longer reported from this Mediterranean island.

### Geographical and Historical context

**Figure 1:**
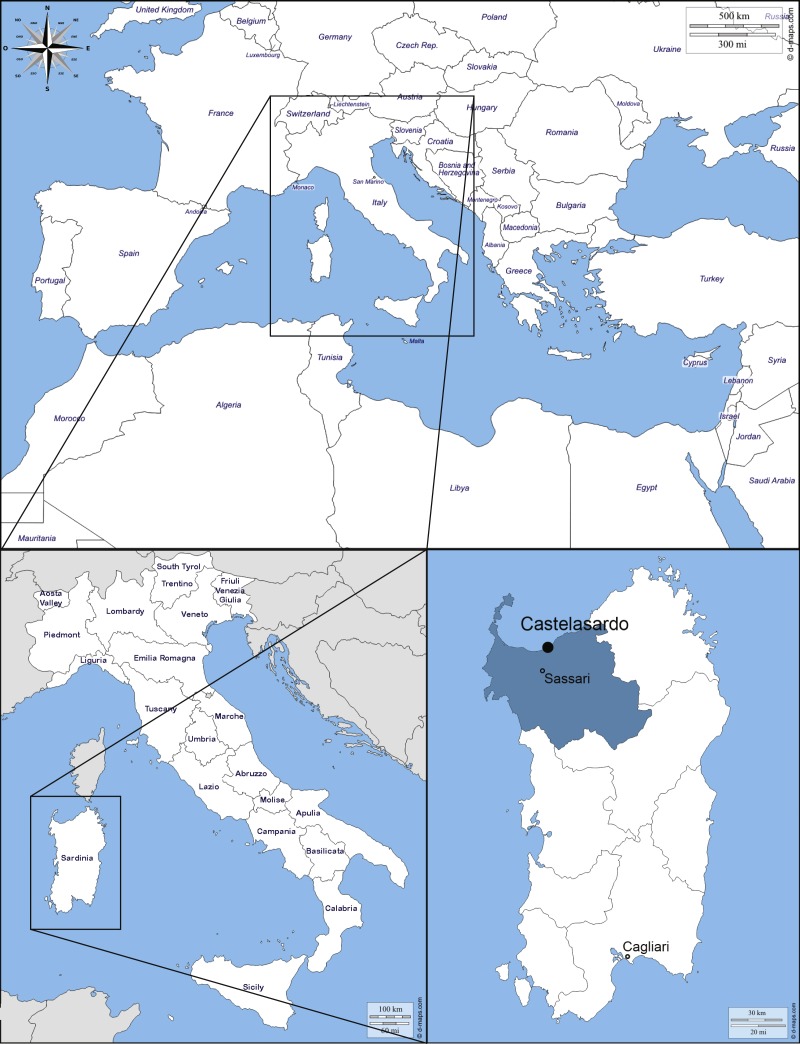
Map of the Mediterranean Basin. The black dot indicates the location of the sampling. Castelsardo is located in the Northern coast of Sardinia (Italy), 114 m asl, as part of Sassari province (40°54 ′52″N8°42′46″E) (maps used for the plate retrieved from: http://d-maps.com/carte.php?num_car=3130&lang=en; http://d-maps.com/carte.php?num_car=4828&lang=en and http://d-maps.com/carte.php?num_car=22838&lang=en).

The small town of Castelsardo is located in the Northern coast of Sardinia (Italy), 114 m asl, as part of Sassari province (40°54′52″N  8°42′46″E) overlooking the Asinara gulf (Fig. 1). The climate is typically Mediterranean and has been classified as Csa type (Köppen & Geiger, 1930; Peel, Finlayson & McMahon, 2007). The average annual temperature is 16.1 °C, with a maximum of 28.9 °C and a minimum of 6−7 °C during the summer and winter seasons respectively (Metereological station of Alghero Fertilia, Sassari, Sardinia; Pinna, 1945). The average annual rainfall is 588 mm (https://it.climate-data.org).

The Sant’Antonio Abate Cathedral, an ancient Romanic Church which became the seat of the bishop of Ampurias in 1503, is located in the old town center. The modern building is the result of the reconstruction which was carried on between 1597 and 1606 due to the transformation of the original Church into Cathedral. Architectonic elements from the Renaissance renovation and original Romanic ones define the style of the Cathedral. The interior structure is based on the Latin cross plan, with a single nave with barrel vaults, side chapels and transept. The old basements *(hypógheios)* of the Church were left unburied and used as sepulchral crypts of the new cathedral.

Modern renovation works began in 2011 to expand the area of the Diocesan museum located in the underground crypts. During the excavations, human remains were found in the so called “Room 3”. Heaps of bones and mummified bodies have been found in different positions and places of the crypt, allowing to reconstruct the relative chronology of the depositions.

Two individuals, conventionally called “Bob and Mary”, are believed to be the most recent inhumations since they have been found at the top of the excavation in close proximity to the floor of the Cathedral. Considering that from 1804 *intra muros* burials were no longer permitted (Napoleon’s Saint Cloud Edict), “Bob and Mary” represent not only the most recent inhumations but probably even the last. At the same time, despite the still uncertainty about the time of the other previous burials, the practice of underground burials in the Cathedral can be dated between 1597 and 1806, when the Saint Cloud Edict was applied in Italy.

The archaeological material was studied by a multidisciplinary international scientific team including archaeologists, anthropologists, biologists, and immunologists in order to investigate about the sanitary condition of the population and to better understand the burial practices carried out in this region.

## Material and Methods

The archaeological excavation of the crypts, that are located under the Sant’Antonio Abate Cathedral in the town of Castelsardo, highlighted the presence of some insect fragments in relationship with the human remains.

Entomological samples were manually collected with sterile tweezers and paintbrushes mainly from the remains of the two individuals previously mentioned but in a context of several burials, and stored in sterile plastic vials. The collection and the study of the archaeological material was authorized by the Ufficio Culturale—Diocesi di Tempio Ampurias in March 2011.

Before microscopic observation Diptera puparia were first carefully cleaned using a solution of soap and water with a wet fine detail paintbrush and then individually sonicated for 30 s using a sonicator bath (QH-Kerry Ultrasonic Limited, *f* = 50 Hz; Kerry Ultrasonics Ltd., Hitchin, UK). Then, the samples were air-dried.

All the samples were observed and photographed using a Keyence VHX-S90BE digital microscope, equipped with Keyence VH-Z250R and VH-Z20R lens and VHX-2000 Ver. 2.2.3.2 software (Keyence, Osaka, Japan).

Sample identifications were performed using specific keys (G Giordani, S Vanin, 2017, unpublished data; Royal Entomological Society of London, 1993; Skidmore, 1985; Smith, 1986; Vienna, 1980) and by comparison with already identified specimens obtained from species breeding. Concerning puparia, the shape and position of the posterior spiracles, anal plate and intersegmental spines were considered as diagnostic characters. Lepidoptera cocoons were identified only at the family level.

## Results and Discussion

Specimens belonging to Diptera, Coleoptera and Lepidoptera were identified among the material (Table 1).

**Table 1:** List of the samples collected from the mummies found in the crypts of the Sant’Antonio Abate Cathedral in Castelsardo, Sardinia, Italy.

	Puparia (full)	Puparia (anterior region with spiracles)	Puparia (posterior region with spiracles)	Puparia (other fragments)	Adults (fragments)	Cocoons
**Diptera**						
nd					3 (legs, wing)	
Muscidae						
*Hydrotaea capensis*	11	2	29	7		
Calliphoridae						
*Phormia regina*	2	1	10	24		
*Calliphora vicina*			31	31		
Sarcophagidae						
*Sarcophaga* sp.			2	1		
**Coleoptera**						
Histeridae						
*Saprinus semistriatus*					2 (elytra)	
Tenebrionidae						
Gen. sp.					1 (elytron)	
**Lepidoptera**						
Tineidae						
Gen. sp.						2

### Diptera

Of the total number of entomological fragments, 95% were Diptera puparia, all of them sampled in the area where the bodies of “Bob & Mary” were located. The number of puparia fragments clearly indicate that this was not a sporadic finding but it is consistent with the colonization by flies in the early phase of decomposition. It was not possible to associate the puparia from one body or the other because of the archaeological context, and because post-feeding maggots tend to move away from the body to find a protected place where the pupariation and later the pupation take place (Lewis & Benbow, 2011; Martín-Vega, Hall & Simonsen, 2016).

**Figure 2:**
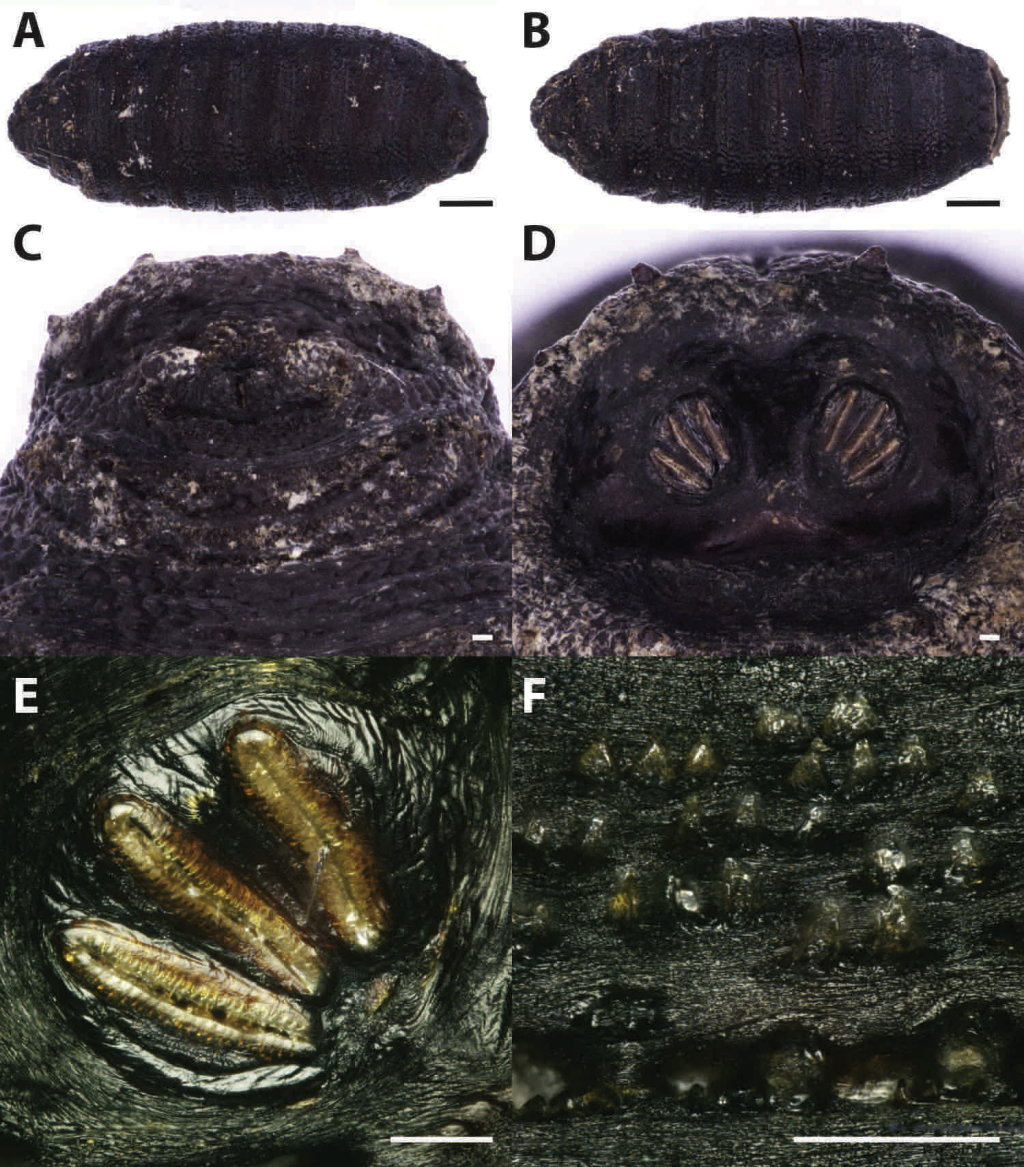
Phormia regina. Puparium in ventral (A) and dorsal (B) view (Black scale bar: 1 mm). Puparium details: anal plate (C), posterior spiracles (D, E), segmental spiculae (F) (White scale bar: 100 µm).

**Figure 3:**
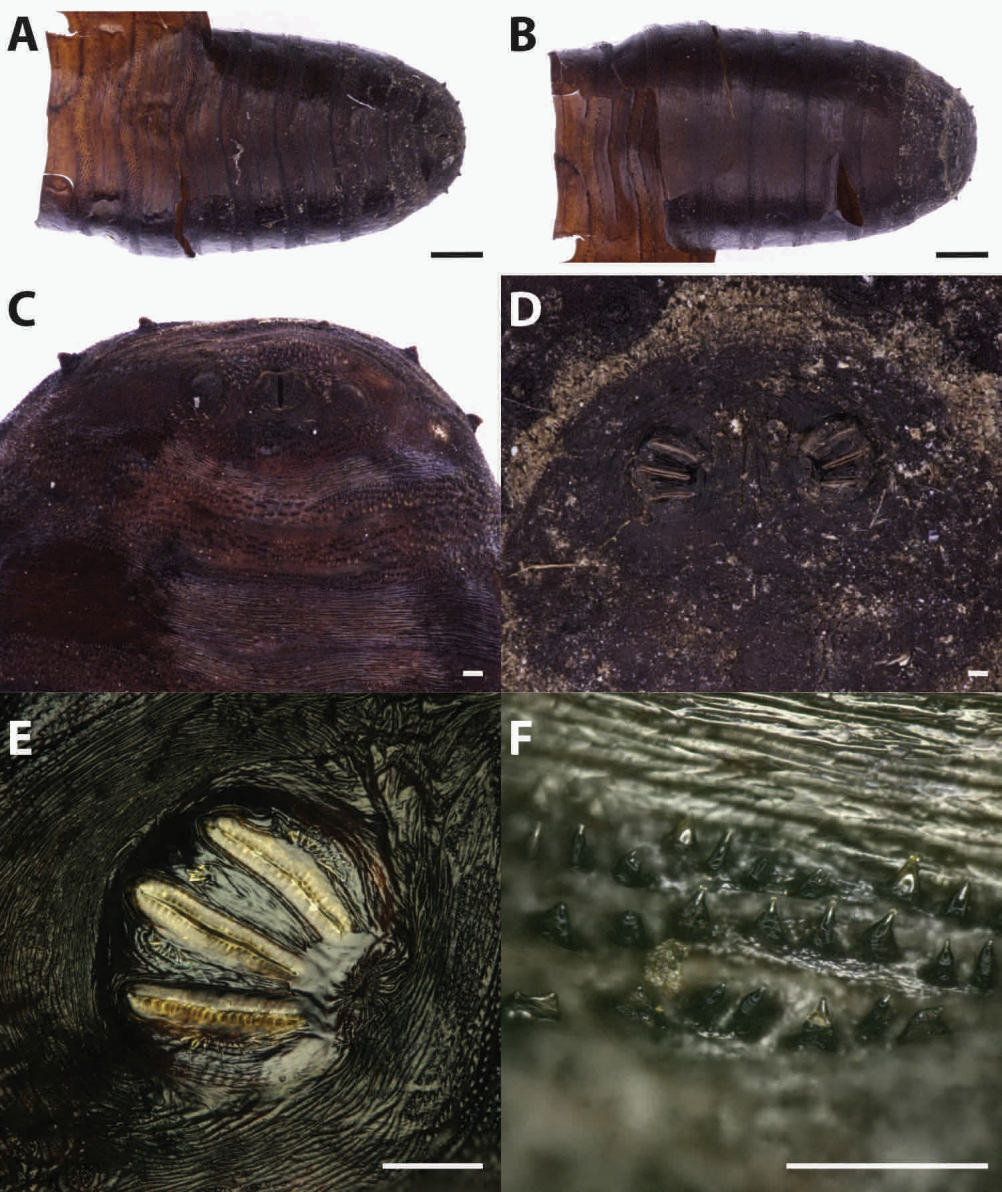
Callihpora vicina. Puparium in ventral (A) and dorsal (B) view (Black scale bar: 1 mm). Puparium details: anal plate (C), posterior spiracles (D, E), segmental spiculae (F) (White scale bar: 100 µm).

Puparia of four different morphotypes were isolated among the studied material: the largest three morphotypes were recognized as members of the families Calliphoridae (Calliphorinae and Chrysomyinae) and Sarcophagidae, the smallest belonging to Muscidae. From the morphology of the posterior region, the shape and position of the posterior spiracles, the structure of the anal plate and the shape and disposition of the intersegmental spines, Calliphoridae puparia were identified as *P. regina* (Fig. 2) and *Calliphora vicina* (Robineau-Desvoidy, 1830) (Fig. 3). Both species are typical of the first body colonization wave and are reported mainly from exposed bodies (Smith, 1986).

*Phormia regina* puparia show a rough appearance and a characteristic posterior region with the posterior spiracle, big and rounded, located in a superficial invagination (Fig. 2), and minute, sharp intersegmental spines arranged in short rows. The lack of big and robust anal papillae with a crow shape is useful for distinguishing this species from *Protophormia terraenovae* (Robineau-Desvoidy, 1830) which has been often reported in association with *P. regina* (e.g., Vanin et al., 2009).

Classified within the subfamily Chrysomyiinae, *P. regina* is a forensically important fly. This widespread, hemisynanthropic^1^ 1Hemisynantropic species usually thrive on the edges of towns, with greater or lesser affinity to humans.species is associated with decomposing material (dung and carrion) (Byrd & Allen, 2001; Greenberg, 1985; Nabity, Higley & Heng-Moss, 2006). In temperate regions, this species is abundant in spring and summer whereas its presence decreases in cooler areas (Goddard & Lago, 1985; Greenberg, 1985; Hall, 1948). Relatively tolerant to cold weather, the minimum and maximum thresholds for this species range from 10–12.7 °C to 35 °C (Deonier, 1942; Haskell, 1993). Rare nocturnal oviposition has been recorded in natural environments (Berg & Benbow, 2013). This species is reported from almost the whole continental Europe, with the exception of the Iberian Peninsula, the Denmark, the Hellenic Peninsula and the Italian main islands (Sardinia and Sicily) (http://www.fauna-eu.org). If this lack of reporting the species from Sardinia is due to a real absence or a misidentification with *P. terraenovae* remains to be evaluated. The finding of this species is particularly important because no modern records of the species are available in the specific literature from Sardinia neither modern specimens from this region are stored in museum collections in Italy. In contrast the species is widespread in Italy, mainland, where it was reported from archaeological and forensic works (Vanin et al., 2008; Vanin et al., 2009; Vanin et al., 2011; Benelli et al., 2014). It is worth mentioning that an important part of the past Sardinian economy was based on the sheep farming and that *P. regina* was reported as cause of myiasis on livestock in North America; however, this species was never been reported from goat and sheep in Europe (Hall & Wall, 1995; Sotiraki & Hall, 2012).

*Phormia regina* was reported from different forensic context in North America and Central Europe (Italy, Germany, Poland, etc.) both on animal carcasses (pigs and fishes) and human bodies (Anton, Niederegger & Beutel, 2011; Cianci & Sheldon, 1990; Goddard & Lago, 1985; Greenberg, 1985; Matuszewski et al., 2008; Matuszewski et al., 2010a; Matuszewski et al., 2010b; Matuszewski et al., 2011; Schroeder, Klotzbach & Puschel, 2003; Sharanowski, Walker & Anderson, 2008; Vanin et al., 2008; Vanin et al., 2011). From archaeological contexts it was reported only from the remains of a WWI soldier in the Italian front (North-Eastern Italy) (Vanin et al., 2009) and on pre-historic graves in Canada by Teskey & Turnbull (1979). In both cases, the authors suggested a considerable lapse of time between death and interment (Teskey & Turnbull, 1979; Vanin et al., 2009).

Puparia of *C. vicina* appear smoother and of a lighter brown colour if compared to the previous species (Fig. 3). The ratio between the major dimension of the spiracle and the interspiracle distance allow to distinguish this species from the cogeneric *Calliphora vomitoria* (Linnaeus, 1758). In fact this ratio is <1 (big spiracles, small interspiracle distance) in *C.vomitoria* and >1 (smaller spiracle, bigger interspiracle distance) in *C. vicina* (Emden, 1965).

*Calliphora vicina* is a common forensically important blow fly, belonging to the subfamily Calliphorinae. This species has an almost cosmopolite, very common in urban habitat, closely associated with humans (Wallman, 2001) but also present in hedgerow or woodland habitats (Smith & Wall, 1997a). *Calliphora vicina* was reported over the whole year (Schroeder, Klotzbach & Puschel, 2003), although in regions with hot summer, its seasonal population curve is typically bimodal with spring and fall peaks where the temperatures are around 13–24 °C (Greenberg, 1985; Salvetti et al., 2012; Zumpt, 1965). Because of its promptness in reaching and colonizing the body after death, *C. vicina* is currently considered as one of the most important fly species in forensic entomology (Davies, 1990; Isiche, Hillerton & Nowell, 1992; Smith & Wall, 1997a; Smith & Wall, 1997b). Its presence on dead bodies or animal carcasses has been deeply studied from all around the world (North America, Europe, Asia and Africa) (Bonacci et al., 2009; Bugelli et al., 2015; Dupont et al., 2011; Greenberg, 1985; Keshavarzi et al., 2016; Moemenbellah-Fard et al., 2015). From archaeological contexts, this species was reported from Central Italy, on partially mummified bodies found in a crypt under a Church (Vanin, 2012) in a circumstance similar to the one described in this work. However, in that context some openings (small windows) allowed the insect arrival and body colonization from the external environment. The ability to colonize bodies in ipogeic context is confirmed by the finding of Faucherre and colleagues (1999). These authors reported the presence of *C. vicina* eggs on a human cadaver found in a 10 m deep cave in the Swiss Jura mountains at an altitude of 1,260 m asl. However, in colonization experiments this species showed a limited ability to colonise buried remains, not deeper than 10 cm (Gunn & Bird, 2011) and it is not reported from buried human remains. In general, the presence of this species indicates a colonization of exposed bodies: before their burial or their transfer into a crypt.

**Figure 4:**
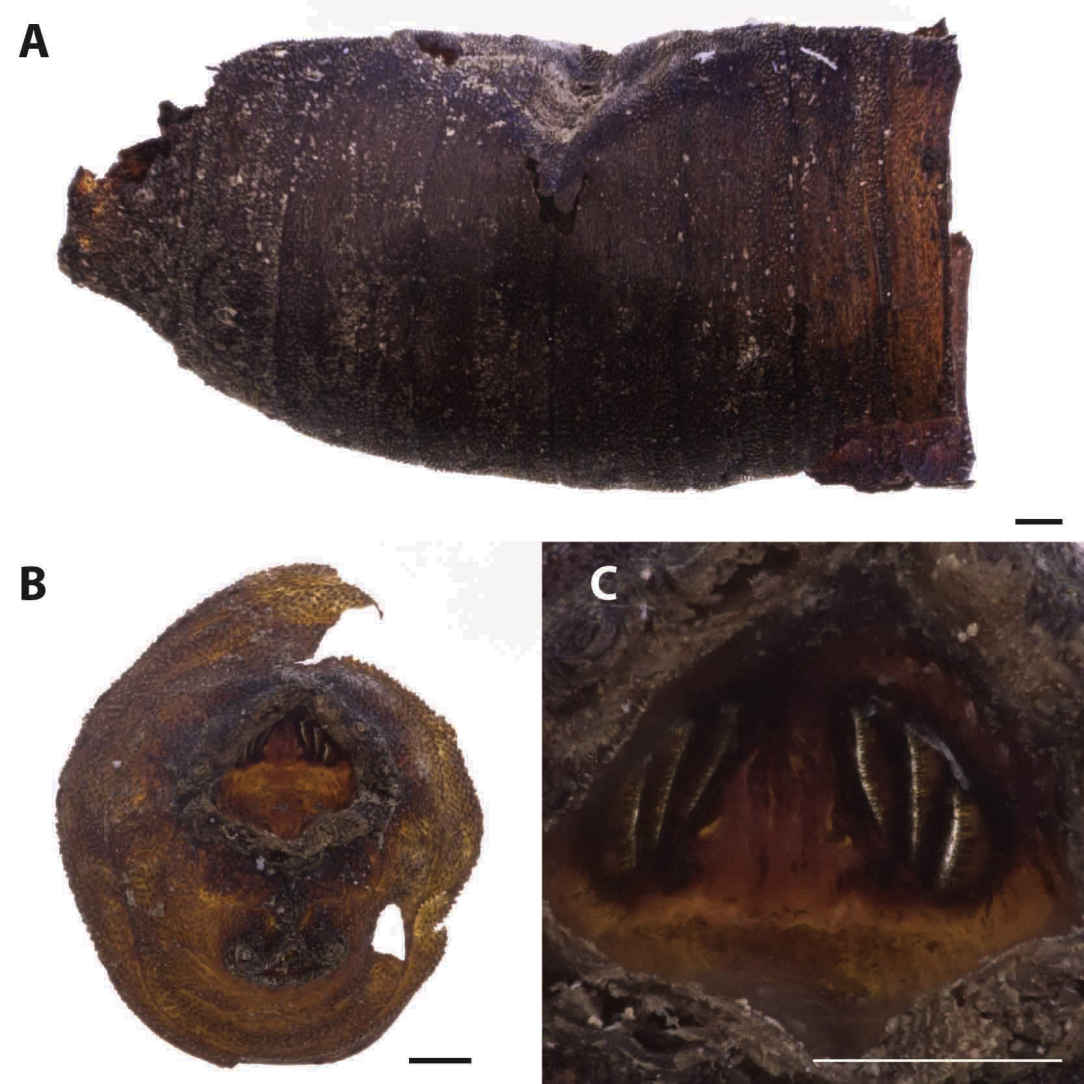
*Sarcophaga* sp. Puparium in later view (A). Puparium details: posterior region with the characteristic crateriform-shaped cavity located in the upper part of the last abdominal segment (B) where the posterior spiracles are located (C) (Black scale bar: 1 mm; White scale bar: 100 µm).

Three puparia fragments (Table 1, Fig. 4) were identified as belonging to a species in the genus *Sarcophaga* Meigen, 1826, in the family Sarcophagidae. Puparia of Sarcophagidae are bigger than the Calliphoridae ones and are characterized by having the posterior spiracles hidden in a crateriform-shaped cavity located in the upper part of the last abdominal segment. The distribution and shape of the spiracle slides and the open peritreme are also diagnostic characters for the family identification (Fig. 4).

Sarcophagidae family includes about 2,600 species worldwide distributed (Pape et al., 2009). *Sarcophaga* females are larviparous and directly deposit first instar larvae on carrions and cadavers. They usually arrive at corpses slightly later than the Calliphoridae and are able to colonize exposed and buried remains (Pastula & Merritt, 2013; Lo Pinto et al., 2017; Bugelli et al., 2015). In Italy the species of this genus are reported being active during the warmer season of the year (Bugelli et al., 2015; Lo Pinto et al., 2017; Vanin et al., 2008). Only few Sarcophagidae records, mainly undetermined species, refer to archaeological contexts (Huchet, 2010; Huchet et al., 2013b).

**Figure 5:**
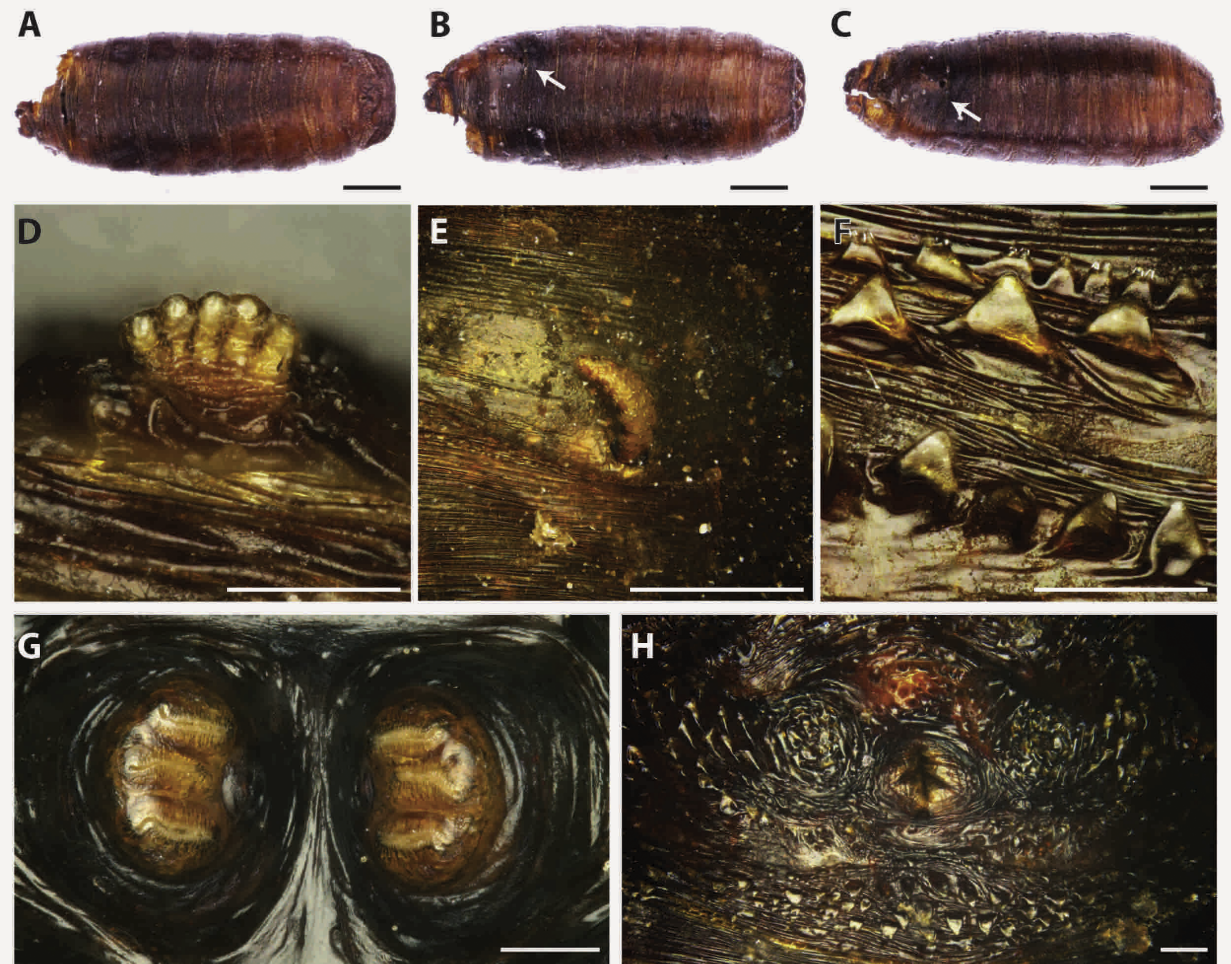
Hydrotaea capensis. Puparium in ventral (A), dorsal (B) and lateral (C) view (Black scale bar: 1 mm). White arrow indicates the position of the respiratory horns. Puparium details: anterior spiracle (D), horn (E), segmental spiculae (F), posterior spiracles (G), anal plate (H) (White scale bar: 100 µm).

The majority of the analysed puparia were smaller than the Calliphoridae ones and showed respiratory horns, a characteristic anal plate and spiracles weakly protruding from the posterior region with three subparallel slits (Fig. 5). All these characters allow the identification of the puparia as belonging to the Muscidae fly, *Hydrotaea capensis* (Wiedemann, 1818). Puparia of *H. capensis* share several features with the very similar puparia of *Hydrotaea aenescens* (Wiedemann, 1830), species introduced in Europe from South America after the XV century and widely distributed in the Mediterranean region. An accurate analysis of the posterior spiracle slits and of the anal plate allows the distinction of the two species (Skidmore, 1985).

*Hydrotaea capensis* is a synantropic species known from a wide variety of habitats, except for the arid ones (Grzywacz et al., 2017). The species was reported from different archaeological and forensic contexts all over the Europe (France, Portugal, Italy, Germany, Spain, etc.). In exposed conditions, *Hydrotaea capensis* is present during the advanced stages of decomposition whereas with buried or concealed bodies it tends to be one of the first to colonize (Greenberg, 1991; Greenberg & Kunich, 2002; Huchet et al., 2013a; Smith, 1986).

In archaeological context the species was reported from mummified bodies in religious burials (Couri et al., 2009; Morrow et al., 2015; Vanin, 2012) and from WWI battlefields (Huchet et al., 2013a).

### Lepidoptera

**Figure 6:**
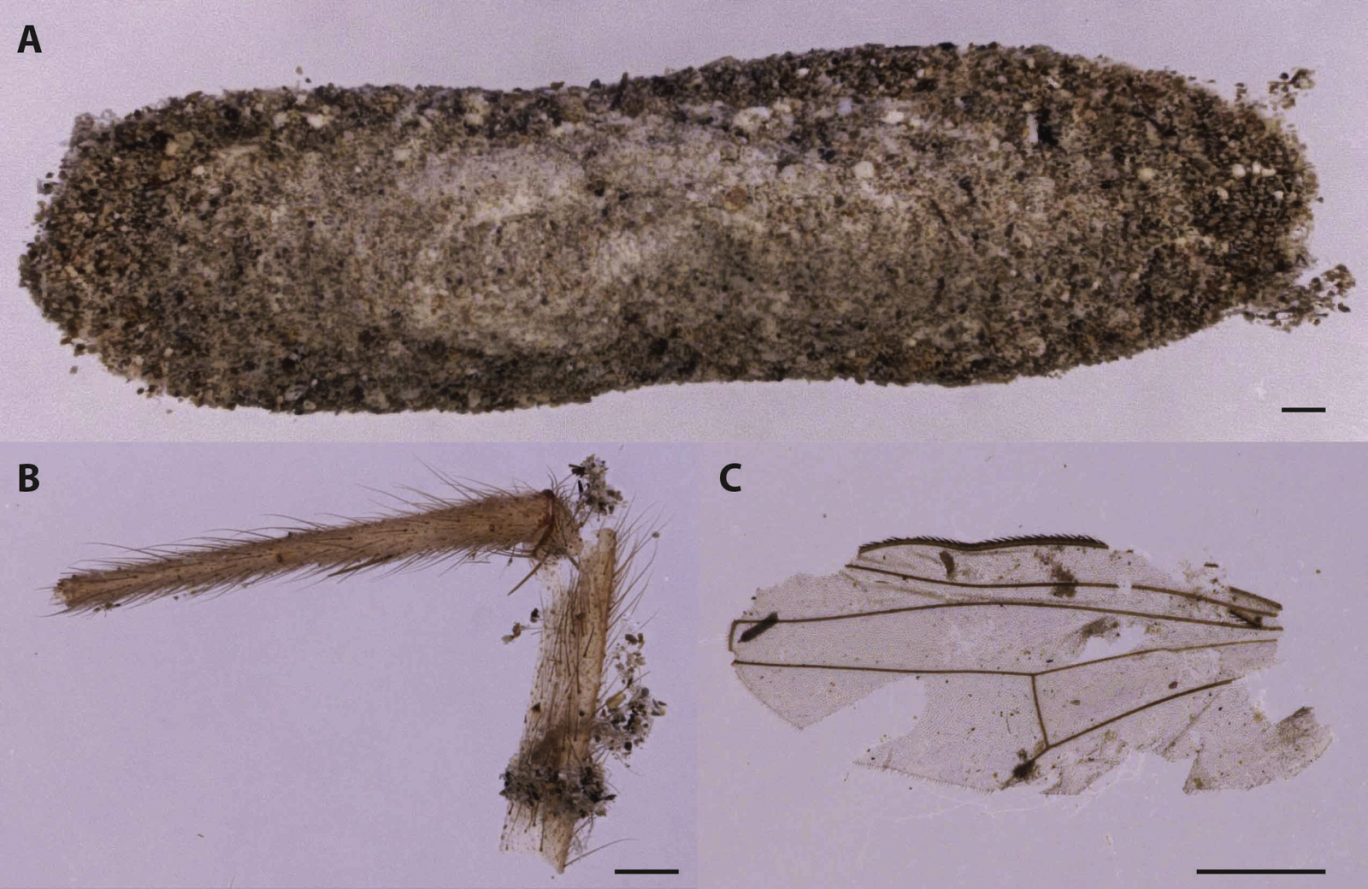
Cocoon of Tineidae moths (A). The cocoon was recovered by moth frass and insect fragments as fly leg (B) and wing (C). This indicates that moth arrival on the human remains was subsequent to the Diptera (Scale bar: 500 µm).

Few moth cocoons were also isolated from the entomological samples (Fig. 6, Table 1). Because of their shape, structure and composition they were identified as belonging to the family Tineidae. The most common species of this family associated with carrion but as well with biodegradation of textiles, with some implications as well in cultural heritage conservation, are *Tinea pellionella* (Linnaeus, 1758) and *Tineola bisseliella* (Hummel, 1823). These species are typical of the last phases of the human decomposition when they can feed on dry tissues and hair (Smith, 1986; Mazzarelli et al., 2015).

### Coleoptera

**Figure 7:**
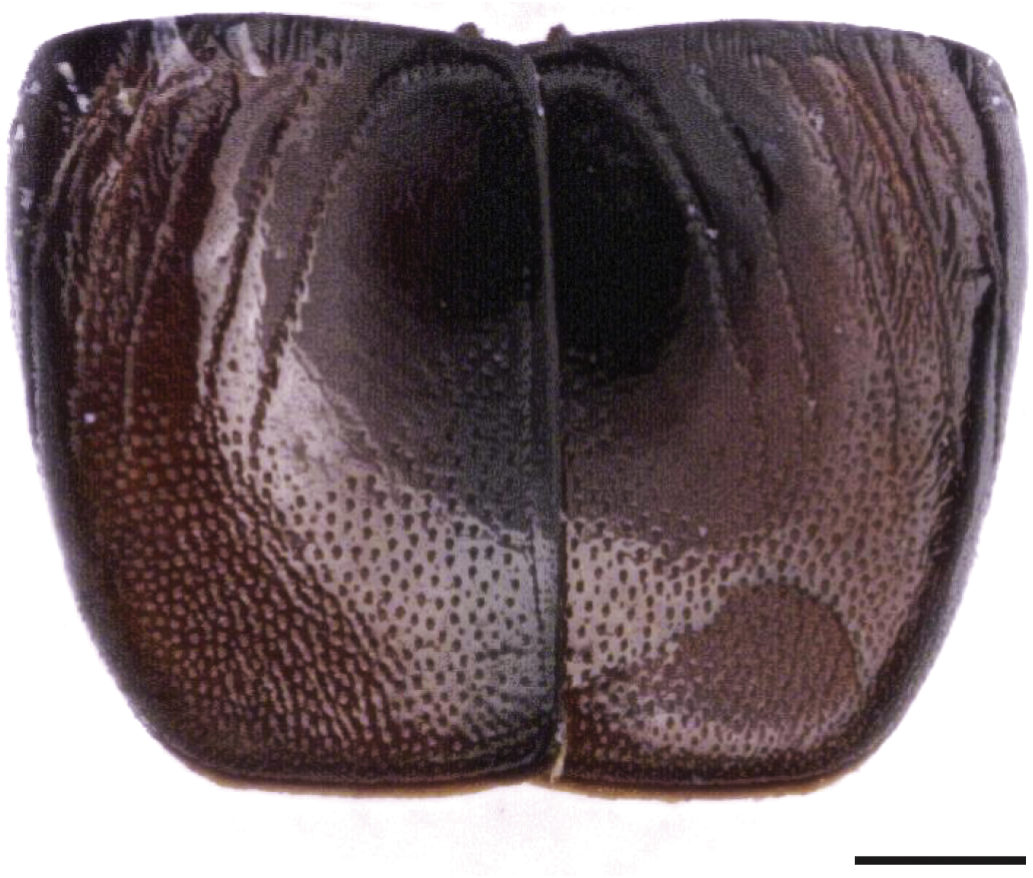
*Saprinus semistriatus.* elytra. (Scale bar: 500 µm).

A single specimen of beetle in the family Histeridae and a single elytron, potentially of a species in the family Tenebrionidae, were also present (Table 1). The hister beetle (Fig. 7) was identified as *Saprinus* (*Saprinus*) *semistriatus* (Scriba, 1790). This species with a Palearctic distribution was reported also from India and Taiwan and is commonly found under animal carcasses and decomposing fishes, manure heaps and rubbish dumps (Vienna, 1980). The species was reported from small and big animal carrion in Europe where it represents the most abundant taxon among Histeridae species during the spring and summer seasons (Bajerlein, Matuszewski & Konwerski, 2011; Kočárek, 2003). In Northern Italy, it was reported from burned and unburned pig carrions during the summer period but not in the winter season (Vanin et al., 2013). Matuszewski & Szafalowicz (2013) found that *S. semistriatus* in addition to *Thanatophilus sinuatus* Linnaeus, 1758, *Necrodes littoralis* (Linnaeus, 1758), *Necrobia rufipes* (De Geer, 1775)*, Necrobia violacea* (Linnaeus, 1758)*, Creophilus maxillosus* (Linnaeus, 1758)*, Philonthus politus* (Linnaeus, 1758)*, Saprinus planiusculus* Motschulsky, 1849 and *Margarinotus brunneus* (Fabricius, 1775) can be used to estimate the pre-appearance interval (PAI)—the time before the appearance of a species on the cadaver/carrion—from temperature. This approach can be used because *S. semistriatus* shows a close relationship between PAI and the environmental temperature.

## Conclusion

The entomofauna collected from an archaeological context does not fully represent the fauna associated with the cadaver during its decomposition: some insects, in fact, move away from the body after their development and others have too fragile structures to survive the taphonomic processes. However, puparia, because of being the result of the feeding stage on the cadaver (larval stage) and due to their immobility, allow a good reconstruction of the diptero-fauna associated with the body decomposition.

Because of their different biology and different ability to reach buried and concealed bodies, Diptera can provide information about the post-mortem events. In this case, as in Vanin et al. (2009), the fauna associated with the body indicates an initial colonization in an open, exposed context, mainly in a warm season (presence of *P. regina* and *Sarcophaga* sp.). The co-presence of *C. vicina* in contrast indicated a colonization in a cooler season, both in exposed condition and inside the crypt, and that specific case could happen inside the crypt after the *P. regina* colonization. *Calliphora* species have been in fact already reported from human bodies found in crypts (Vanin, 2012) and caves (Faucherre, Cherix & Wyss, 1999). A co-presence of the two species cannot be excluded especially at the end of spring when adults of both species could lay their eggs on exposed bodies. In addition, because of the presence of other burials in the same “room” a cross-contamination cannot be excluded.

This research allows the reporting of the presence, at least in the past, of *P. regina* from Sardinia. The list of the species from Sardinian modern field samplings (Rognes, 2011) and the analysis of some Sardinian entomological collections do not indicate the presence of this species in the island. In contrast, the species is reported from the Italian mainland and the other circum-Mediterranean countries (http://www.faunaeur.org). Despite that the hypothesis of a case of local extinction cannot be excluded, further samplings in archaeological context are needed to understand the presence/distribution of the species in Sardinia. Additional research will help in the understanding of the potential reasons of *P. regina* disappearance from Sardinia. This potential “extinction” could be related with the change in the space sharing between people and livestock that occurred in the last century. *Phormia regina*, in fact, has been reported to be a common agent of sheep myiasis in North America and sheep rearing has been an important part of the Sardinian economy.

## Additional Information and Declarations

### Field Study Permissions

The following information was supplied relating to field study approvals (i.e., approving body and any reference numbers): The collection and the study of the archaeological material was authorized by the Ufficio Culturale—Diocesi di Tempio Ampurias in March 2011.

### Data Availability

The following information was supplied regarding data availability: The raw data (pictures of the samples) are included in Figs. 2–7 in the manuscript. All the specimens are stored in the collection of the Forensic Lab for Entomology and Archaeology under the responsibility of Dr. Stefano Vanin.
